# Spectacle lenses for control of myopia progression in children

**DOI:** 10.1007/s44402-026-00149-6

**Published:** 2026-07-31

**Authors:** David A. Atchison, W. Neil Charman

**Affiliations:** 1 The Centre for Vision and Eye Research, Queensland University of Technology, Kelvin Grove, Brisbane, Queensland Australia; 2 The School of Health Sciences, Faculty of Biology, Medicine and Health, University of Manchester, Manchester, United Kingdom

**Keywords:** DIMS, Multisegment, Myopia control, Spectacles

## Abstract

Many new optical treatments have been developed recently to slow the progression of myopia. Most of these are based on theories of how myopia develops, such as poor accommodation, the type of peripheral refraction and image contrast (contrast modulation). Optically related treatments include spectacle lenses, contact lenses, orthokeratology, light therapies, more outdoor time, better interior lighting, indoor illumination, pharmacological intervention (through pupil and accommodation effects) and combination therapies. Current myopia-control spectacle lenses are described, including progressive addition, multisegment, annular cylinder, diffusion optics technology and concentric bifocals. Background is provided and a critical eye is passed over some of the designs. Short-term (12-month) efficacies for both axial length and myopia reduction in myopia-control spectacle wear, relative to single-vision lenses, are about 50%, as compared to 50%, 60% and 80% for contact lenses, orthokeratology and red-light therapy, respectively. Spectacle lens treatments for myopia control are safe, valuable tools in clinical practice. Further laboratory and clinical studies should help to clarify the nature of the mechanisms of action and improve efficacy.

Key points

- Over the last 15 years or so, many new optical treatments have been developed to slow the progression of myopia, most being based on theories of how myopia develops.
- The emphasis here is on spectacle lenses, with background given to some lenses and a critical eye passed over some of the designs.
- Reduced modulation transfer at medium and high spatial frequencies in the peripheral retina may be a common factor in the mechanisms of some treatments; further development should clarify the mechanisms and improve their efficacy.

## Introduction

Myopia is a growing problem in the modern world. Understanding of its causes and development has proceeded slowly and erratically. The basic optics of the myopic eye were finally outlined around 1600 by Kepler and others [1]. In 1813, Ware [2] observed that post-natal conditions and experience must influence myopia development when he found that the refractive error occurred commonly among students and other educated people but was rare among private soldiers or rural workers. This led Donders to remark, “On the distribution of myopia, position in society has a great deal of influence” [3], although he implied that genetic factors were also involved by pointing out that myopic families occurred among all occupational groups. His conclusion, as well as that of Helmholtz, was that the stresses on the eyeball caused by accommodation, convergence and poor posture associated with the use of short working distances and downward gaze during prolonged near work were the prime causes of most myopia [3–5]. These ideas were accepted broadly among the ophthalmological community, although in 1913 Steiger [6] was still suggesting that refractive errors occurred because corneal refraction and axial length were unrelated, freely variable parameters, so that the full range of refractive errors could be explained by the chance combination of these two factors.

For most of the 20th century, attempts to control myopia development in both adults and children were dominated largely by methods designed to decrease the accommodation/convergence demands of near work using bifocals or lens–prism combinations. Although modest success was claimed in selected sub-groups of children, e.g., those who were esophoric at near, in general results were disappointing [7].

Towards the end of the 20th century, two groups of studies encouraged new approaches to slowing myopia progression in young eyes. In the first group, Hoogerheide and colleagues [8] claimed that there appeared to be an association between the relative peripheral refraction, i.e., the difference between the central and peripheral mean refractions, and the development of myopia. They found that initially emmetropic trainee pilots who had relative peripheral hyperopia were more likely to develop axial myopia during their training than those with relative peripheral myopia. In the second, larger group of studies, researchers using animal models showed that placing a positive lens in front of the young eye made the eye develop compensatory hyperopia and a shorter axial length, whereas a negative lens gave a longer, myopic eye. In other words, inducing myopia slowed eye growth, and inducing hyperopia increased eye growth. In both cases, distant objects were approximately imaged on the retina by the lens–eye combination [9]. Thus, it was clearly demonstrated that eye growth could be influenced by an external lens and hence that eye growth was visually regulated. Further animal studies showed that bifocal spectacle lenses, which nominally affected only peripheral imagery, could influence central refraction [10–14]. On the basis of these and other concepts, new designs of spectacle and contact lenses have emerged, which aim to slow the progression and reduce the final level of myopia in children and adolescents. Orthokeratology, which modifies the corneal shape and hence the eye’s peripheral optical aberrations, plays a similar role [15].

Much of this background material has been covered in earlier virtual and special issues of “Ophthalmic and Physiological Optics” [16–18], and an editorial by Bullimore [19] gave a useful summary of the situation in 2024. In the present review, we discuss the developments of these and related ideas that have occurred within roughly the last 15 years. Topics covered include the theoretical background to methods of myopia control, the designs of spectacle lenses that are now available, the success that lenses have achieved and how this compares with other optical treatments of contact lenses, orthokeratology and red-light therapy, and the degree to which the mechanism of their action is understood. This is not an epidemiologically based review and does not include critical evaluations of the methodology of clinical trials or the efficacy of the lenses in each cited trial. At present, comparisons of published values of efficacy may be misleading due to differences in the protocols and patient samples used in trials of the different treatments.

## Theories concerning onset and progression of myopia and relevance to optical correction of myopia

We list here several current hypotheses concerning the factors that might lead some children to develop myopia rather than maintain the state of near-emmetropia that is usually achieved in the first few years of life. Wider summaries, including additional possible mechanisms, have recently been provided by Schaeffel et al. [20] and Schaeffel and Swiatczak [21]. Most of the newer treatment methods that will be discussed later were originally based on the Peripheral refraction, Simultaneous vision and Image contrast theories below and depend upon the modification of the images falling on the mid-peripheral retina [12]. We have not discussed genetic factors, since at present these seem unable to explain the recent rapid increases in prevalence of myopia [22], but it may be that future work will reveal important interactions between genetic and modern environmental factors [23].

### Excessive or prolonged accommodation

As mentioned previously, Donders and Helmholtz believed that stresses on the eyeball caused by accommodation, convergence and poor posture were prime causes of most myopia. This idea persisted in the scientific literature until at least the 1970s [24], and still has many supporters who link the rise in childhood myopia to factors such as the much greater levels of close work in current education, widespread use of hand-held screen devices which demand close observation distances and a reduction in the time that children spend outdoors [25–27]. Non-optical treatments based on these assumptions have included high-dose atropine to paralyse accommodation, minimising the time spent in close work, improved school lighting [28], the introduction of longer periods of outside play, reduction in screen time and similar measures [25]. Optical treatment could involve additional positive power lenses to reduce the stimulus to accommodation and/or convergence.

### Poor accommodation

According to most studies on the matter, children who develop myopia or already have myopia tend to have poorer accommodation responses than emmetropes [29]. It has been suggested that the consequent hyperopic defocus during near vision tasks stimulates eye growth. Optical treatment involves compensating for the poor response by additional positive power (or alternatively, less negative power) in the central field; this was the main method in the second half of the last century and into this century. To a considerable extent, this is opposed to the previous theory, although one of the treatments is the same!

### Mechanical tension

It is claimed that mechanical tension caused by the eye lens or ciliary body restricts equatorial eye expansion and, in turn, this results in abnormal eye growth and axial elongation; certain factors accelerate this in at-risk eyes [30]. If this is true, this theory does not appear to be amenable to optical treatment.

### Peripheral refraction

The hypothesis here is that relative hyperopic peripheral refraction stimulates eye growth. Optical treatment involves compensating for the relative refraction by additional positive power (or less negative power) in restricted zones or across the peripheral field.

In 1971, Hoogerheide et al. [8] indicated a link between relative peripheral hyperopia and myopisation. This was picked up in 2004 by Wallman and Winawer [31] and Stone and Flitcroft [32], and has since become a mainstream theory of myopia onset. The general understanding is that Hoogerheide et al. reported peripheral hyperopia along the horizontal visual field of young emmetropic adults that was associated with myopia development. However, some detective work forty years later made it clear that peripheral refraction was often taken well after these emmetropes did, or did not, develop myopia [33]. That this was not reported by Hoogerheide et al. was probably because they considered the peripheral refraction pattern to be innate: “It may be presumed and certain indications do exist that the general appearance of the skiagram [the peripheral refraction pattern] is inborn and does not change very much during lifetime.” As mentioned in the Introduction, animal studies show that the eye can grow in response to peripheral stimuli without the involvement of the fovea. Mutti et al. [34] measured peripheral refraction at the 30° nasal visual field and found some evidence that relative peripheral refraction developed some time before myopia onset in children, but later clinical studies found that relative peripheral hyperopia along the horizontal visual field does not occur until after myopia development, i.e., it is a consequence, rather than a cause, of, myopia development [35–37]. Another relevant factor is that most emmetropes and myopes do not have relative peripheral hyperopia along the vertical field meridian, and it would seem unlikely that peripheral refraction would drive myopia development only along and adjacent to the horizontal field.

There have been further studies on the predictive effect of relative peripheral refraction upon the development or progression of myopia, but none have supported a firm link between relative peripheral hyperopia and myopisation. Rotolo et al. [38] confirmed the findings of the previous studies [35–37]. Leighton et al. [39] found that greater hyperopic relative peripheral refraction in the temporal visual field of myopic children was “indicative of increased risk for rapid axial elongation”; this cannot, however, be considered to be conclusive, as the refractions of the slower and faster growing eyes were not indicated. In an initially emmetropic group of children, Lin et al. [40] found that a sub-group who showed greater central refraction change had greater *myopia* initially in the superior retina; they believed this to be predictive of myopia development. They suggested that this was because this region was becoming myopic before the central retina rather than being a driver for myopic development. Xie et al. [41] found that differences in peripheral refraction between emmetropic and low myopic groups were considerable, and claimed that measuring peripheral refraction may predict myopia onset, but without supporting longitudinal data, such a claim is not warranted.

Some researchers have considered the possibility of treatment changing retinal shape and, in particular, asymmetry [42].

In spite of these doubts about the role, if any, of relative peripheral hyperopia, the use of positive additions in the peripheral field obviously slows myopia progression (see the section “Treatment success” below). This makes it likely that these peripheral refractions affect some other visual quality parameter, which then drives axial elongation. In this context, it is worth mentioning a recent lens with two variants, one of which has additional negative power in the periphery and which works as well as the lens with additional positive power (see the sections “Myopia control by spectacle lenses, Multisegment lenses and Lenslet-ARray-Integrated (LARI) lens”) [43, 44].

### Simultaneous vision (dual-focus) theory

Experiments on chicks show that the growing eye responds to the presence of a positive lens by becoming hyperopic [9]. Moreover, if the lens has alternating zones of differing power or sign, the final refractive state of the eye represents a compromise between those expected from each lens section alone [45]. This suggests that eye growth and myopia development could be slowed if the retina were provided with both the normal retinal image and a second image in a myopic image plane. This additional image might be produced either by adding small, positive, refractive elements to the surface of a spectacle lens, the size of the elements being small enough to ensure that several lie within the imaging beams or by contact lenses with a suitable radial or other variation in power. An extension of these ideas, strongly advocated by the developers of the EssilorLuxottica Stellest (Essilor.com) multisegment spectacle lenses (see below), is the concept of generating a “volume of defocus” rather than distinct retinal and myopic foci. This can be achieved by making the additional refractive elements strongly aspheric.

### Image contrast (contrast modulation) theory

The hypothesis here is that the high contrasts found in outdoor and indoor urban environments stimulate eye growth and the development of myopia [46]. This is susceptible to genetic factors.

A sophisticated explanation has been developed based on ON- and OFF-midget bipolar cells [47, 48], which act as contrast detectors so that their activity drives axial growth; lowering contrast reduces activity. The explanation includes a role for genetics, with defects in the genes that control colour vision leading to signalling of increased contrast at the bipolar layer. One variant of these ideas is that the spatial frequency content of man-made environments differs markedly from that in natural environments, and has characteristics similar to those which have been shown to induce form-deprivation myopia in animal models [49]. It is interesting that this theory contradicts Wallman’s hypothesis [31] of ocular development that retinal activity, such as obtained with high-contrast images, inhibits eye growth.

### Other aberrations

Aberrations other than the peripheral refraction components, and in particular spherical aberration, have occasionally been suggested as being involved in the progression, or prevention of progression, of myopia. Positive spherical aberration can be added with aspheric surface contact lenses [50]. As well as an increase in power towards the edge of the optical area, these lenses will add additional positive power for peripheral vision, thus inducing relative peripheral myopia, which is the recommended treatment according to the Simultaneous vision and Image contrast theories. Animal studies indicate that chromatic signals through longitudinal chromatic aberration appeared to be involved in emmetropisation [51].

## Optical interventions for myopia control—general

**Fig. 1:**
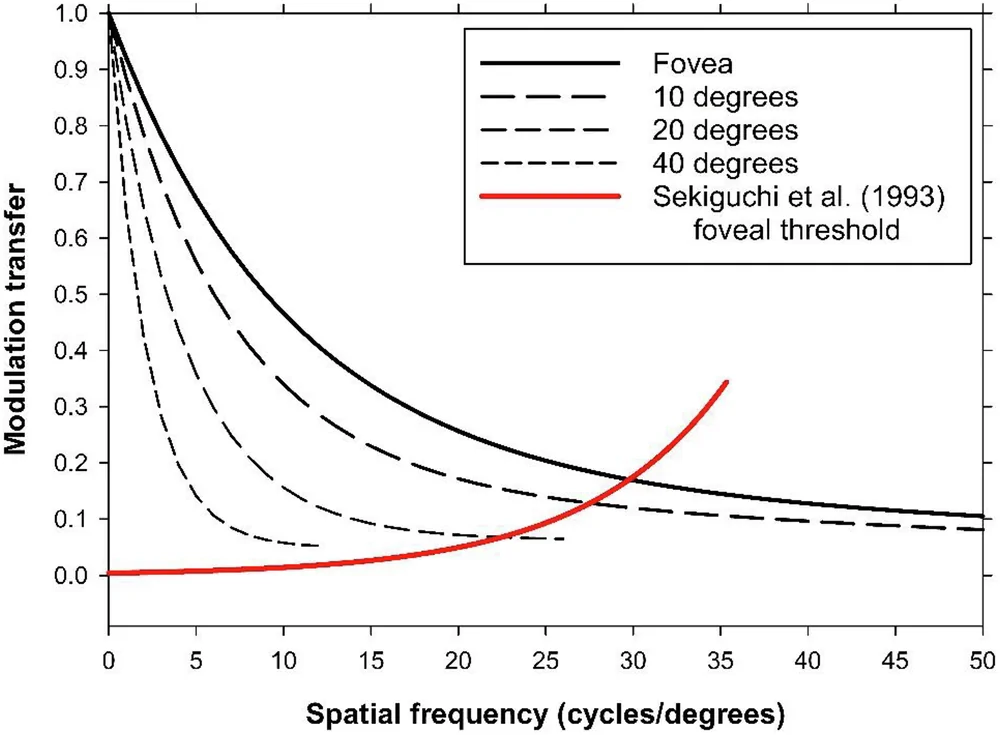
Typical modulation transfer functions for the adult eye with a 3 mm diameter entrance pupil as a function of field angle, with correction for defocus but not astigmatism [53]. Also shown are retinal contrast thresholds for the foveal (on-axis) case from Sekiguchi et al. [54].

Several of the optically related corrections for myopia involve manipulation of the optical image that falls on the peripheral retina. When considering the possible effects of these myopia-control interventions, it must be remembered that, in normal eyes, optical performance deteriorates as the field angle gets larger, due to rising levels of chromatic and monochromatic aberrations, particularly oblique astigmatism, e.g., Lundström and Rosén [52]. Figure 1 shows some typical experimental ocular modulation transfer functions (MTFs) at different field angles [53] together with results for the foveal photopic contrast threshold at the retinal level [54]. Note that the predicted foveal resolution level for high-contrast sinusoidal gratings, as given by the intersection of the foveal MTF and the threshold curve, is about 30 cycles/degree. MTF and neural sensitivity both fall with angle away from the fovea to give a resolution cut-off of about 3 cycles/degree at 30° field angle [55]. In general, an optical intervention that affects only the high spatial frequency content of the peripheral retinal image may produce no visual effect if the retinal sensitivity for these frequencies is inadequate.

## Myopia control by spectacle lenses

As discussed in the “Introduction” section, spectacle lenses have a history of about 60 years in the treatment of myopia [56]. Initially, bifocal or lens-prism combinations, and later conventional progressive lenses, were used to decrease the accommodation/convergence demands of near work. Current lenses of this type are covered in an earlier review [56]. Now, commercially available lenses will be considered, which have become available in the last decade, which depend, at least nominally, upon imagery in the retinal periphery and the hypotheses on myopia development outlined earlier. Table 1 provides a summary of these lenses.

**Table 1:** Recent myopia control spectacle lenses.

Lens grouping^a^	Study/references	Name, description
Progressive addition lenses developed for myopia treatment	Tarutta et al. 2015, 2018, 2019 [58, 61, 62] Silva-Leite et al. 2023 [59] Papadogiannis et al. 2023 [60]	*ArtOptica Perifocal* Horizontal field addition zones with up to 2.5 D addition nasally and 3.0 D addition temporally.
	Rodenstock [63]	*Rodenstock MyCon* Horizontal addition zones
	Carl Zeiss Vision [65, 125] Kanda et al. 2018 [67]	*Carl Zeiss MyoVision/Pro, and MyoVision Ace* Designed for the management of peripheral defocus and have central distance zones surrounded by functional zones of additional positive power
	Yeo et al. [68]	*Essilor Myopilux Lite and Myopilux Plus* are similar to conventional progressive power lenses
	Yuval et al. 2023 [69]	*Shamir Optimee (Shamir Myopia Control)* U-shaped clear distance zone extending mainly superiorly, surrounded by the peripheral addition
	Sánchez-Tena et al. 2024 [70] Martinez-Perez et al. 2025 [71]	*Indizen Optical Technologies Asymmetric Myopic Peripheral Defocus Lens (MPDL)—also called “MyoLess”* Similar to Shamir Optimee
Multiple segments	To et al. [73, 74] Lam et al. 2020, 2022, 2023 [75, 76, 82] Zhang et al. 2020, 2022, 2023 [77–79] (Selection of patents and papers)	*Hoya MiyoSmart (DIMS)* Uses “defocus incorporated multi-segment” (DIMS) technology Distance central zone of 9 mm surrounded by lenslets arranged in 9 hexagons Lenslet diameter 1.0 mm, addition +4.0 D
	Tse et al. 2026 [94]	*Hoya MiyoSmart iQ (also DIMS TED)* Developed from MiyoSmart, Distance central zone 7 mm, addition +4.0 D, treatment zone 40 mm.
	Opticare [95]	*Shanghai Conant Optics MyoEase* Regular matrix of lenslets, hexagonal outer boundary
	Chen et al. 2024 [96]	*Shanghai Weixing Optical Co. Defocus distributed multipoint (DDM)* Additional power increases outwards from +3.5 to +4.5 D.
	Lu et al. 2025 [97]	*Fitlens Medical Technology Co Diversified Segmental Defocus Optimisation (DSDO) Aura 3 and Aura 6* Lenslets arranged in 32 radial rows, additional power reduces outwards from +4 to +2.5 D
	Bao et al. 2022, 2022 [99, 100] Gao et al. 2021, 2023 [101, 165] Sandaridurg et al. 2023 [102] Li et al. 2026 [166] (selection of papers)	*Essilor Stellest* Distance central zone of 9 mm surrounded by lenslets arranged in 11 rings, with adjacent lenslets touching Lenslet diameter 1.1 mm, addition in lenslet centre varies between rings (5.8 D inner to 3.5 D outer)
	Raveendran et al. 2025 [103]	*Stellest 2.0 (H.A.L.T. Max)* Lenslet powers and asphericities are about twice that of Stellest
	Wang et al. 2025 [114]	*Mingyue Optical Lens Co., Ltd. Asymmetric Multipoint Defocus Technique (AMDT) lens* Triangular-like distance zone, 1329 lenslets of +5.0 D power in ring pattern. Lenslets inside the lens rather than on the surface
	Xia et al. 2023 [115] Jiang et al. 2025 [116] Su et al. 2024, 2025 [43, 44]	Shanghai iSparX Medical Lenslet-ARray-Integrated (LARI) lens (Hexagonal lenslets. PLARI has +3.0 D addition and NLARI has −3.0 D addition.
	Wu et al. 2024 [118] Kou et al. 2025 [119]	Shenzen Thondar Individualised ocular refraction customisation (IORC) lens Back surface designed to correct relative peripheral refraction of individual patients. Lenslets in rings 9 to 38 mm diameter. High, medium and low additions (IORC-H, 4.5 D; IORC-M, +3.5 D; IORC-L, +2.5 D)
Annular Cylinder lenses	Carl Zeiss [65, 125] Liu et al., 2023 [120] Alvarez-Peregrina et al. 2023 [121, 122] Chen et al. 2025, 2025, 2026 [123, 124, 167]	*Carl Zeiss MyoCare and MyoCare S* Additional power arranged in ~30 cylindrical rings of 0.5 mm diameter each MyoCare”—children younger than 10 years, central distance zone of 7 mm and mean addition (half the added cylindrical power) of 4.6 D MyoCare S—older children, central zone 9 mm, addition +3.8 D.
	Lin et al. 2026 [168]	*Carl Zeiss MyoEase/Tamer* Four cylindrical rings of about 1.3 mm, and the main lens has additional positive power in the periphery
		*Carl Zeiss MyoActive* Looks like MyoCare lenses, but the power varies around rings and the powers in adjacent parts of the ring do not match.
Diffusion optics technology (DOT)	Neitz and Neitz 2017 [130] Rappon et al., 2023 [129] Laughton et al. 2024 [131] Hill et al., 2026 [169]	*SightGlass Vision Diffusion Optics Technology (DOT)* Clear distance zone of 5 mm diameter Diffusers/scatterers have “a diameter of ~0.14 mm and a height of ~0.2 mm, with an irregular radial curvature that is steeper on the sides and flattened across the top.” [129] Spacing is about 0.3 mm.
	Internacional Optica [133] Szeps et al., 2023 [134] De Tomas et al. 2024 [170]	*International Optics—Diffusion Optics Multiple Optics (DOMS)* Central 9 mm distance zone, surrounded by 1.0 mm scattering dots, separation 0.75 mm Dots extend out to 36 mm in diameter.
Concentric bifocals	Novar Technologies [136] Iribarren et al. 2026 [137]	*Novar Technologies MYOFIX* Central 9 mm distance zone, short transition zone and ~+3.50 D add for the rest of the lens
	OptiCare [95]	*OptiCare MyOnic* Distance zone of 10 mm, surrounded by a transition zone whose power increases to +2.00 or +3.00 D, and after which the power is constant

^a^In all listed trials for which it is relevant, there was a single vision control group.

The spectacle lenses are divided into progressive-addition lenses developed for myopia treatment, multisegment lenses, annular cylinder lenses, DOT Technology and concentric bifocals. Diagrams for several of these are given by Atchison and Charman [56]. Previously, the Zeiss MyoCare lens (zeiss.com) was included under multisegment lenses, but is categorised separately here as an annular cylinder lens. While the lenses of which we are aware have been included, at the time of writing, there may be at least 600 such lenses available in China alone (Stephen Vincent, personal communication).

### Progressive-addition (relative myopic peripheral refraction-based) lenses

Adoption of the relative peripheral refraction theory led to the design of progressive lenses in which the additions are placed at locations other than the conventional position below the distance section. Such lenses date back to about 2009, when the effectiveness of lenses with a radial power gradient was first explored [57]; since then, manufacturers have produced designs with a variety of symmetric and asymmetric refractive gradients.

The ArtOptica Perifocal lens (perifocallens.com) has been available since 2011. Progressive addition is restricted to the horizontal meridian, with up to 2.5 and 3.0 D additional powers provided in the nasal and temporal field regions, respectively. Naturally, it reduces peripheral relative hyperopia along the horizontal visual field [58–60]. Wearer trials have been conducted in Russia and Spain, with data available for 4–5 years [61, 62]. The Rodenstock MyCon lens (rodenstock.com), released in 2023, also provides additional positive power in the horizontal visual field [63].

Carl Zeiss Vision (zeiss.com) has MyoVision/Pro and MyoVision Ace lenses [64–66] which provide central distance vision, surrounded by areas of additional positive power. In a 2-year trial, Kanda et al. [67] found no significant differences in terms of myopia progression and axial length elongation between a group of children wearing the MyoVision lens versus a single-vision control group.

EssilorLuxottica (essilor.com) offers the Myopilux Lite and Myopilux Plus lenses. Power plots for these resemble conventional progressive addition lenses, with “Lite” having a +2.00 D addition and the “Plus” lens having a wider near zone than the “Lite” version [68].

There have been no recently reported clinical trials of the above Carl Zeiss and Essilor lenses. The treatment success of the newer Zeiss MyoCare and Essilor Stellest lenses (see below) may result in production of these earlier lenses being discontinued.

**Fig. 2:**
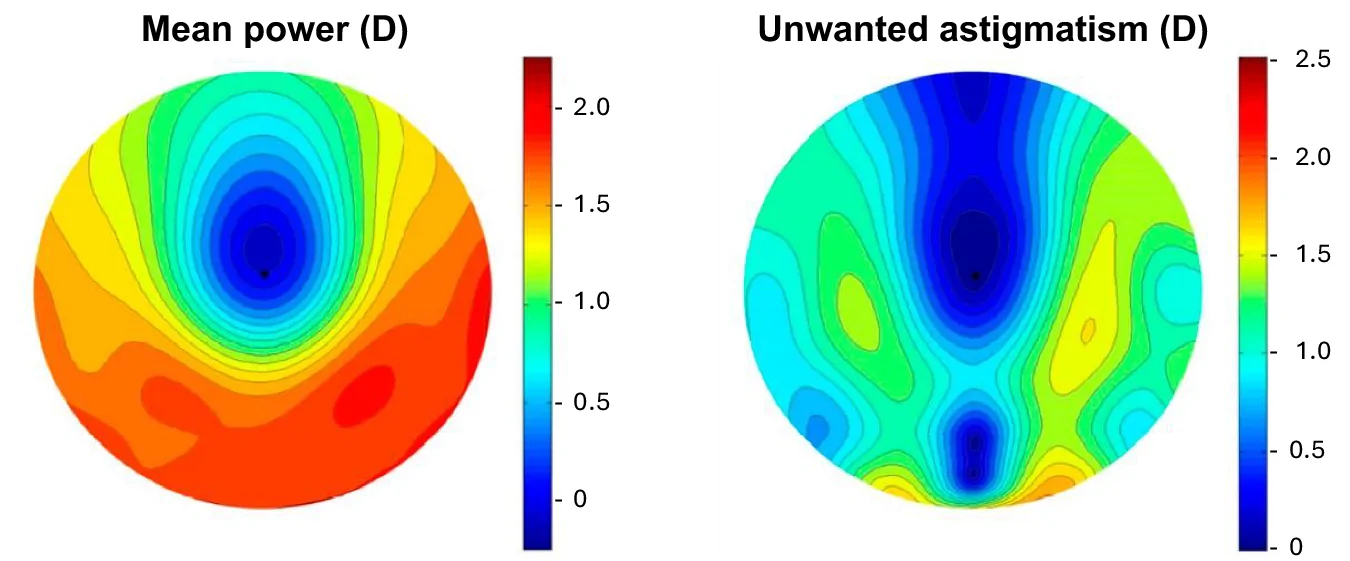
Mean power and unwanted astigmatism of a plano distance Myopic Peripheral Defocus Lens (MPDL) lens. Modified from Sánchez-Tena et al. [70].

The Shamir Optimee Myopia Control lens (shamir.com) was described in a 2024 clinical trial report [69]. The U-shaped distance refraction zone is surrounded by the peripheral addition. Furthermore, the asymmetric myopic peripheral defocus lens (MPDL, iotlenses.com) [70, 71] appears to be similar in its power distribution (Fig. 2).

### Multisegment lenses

**Fig. 3:**
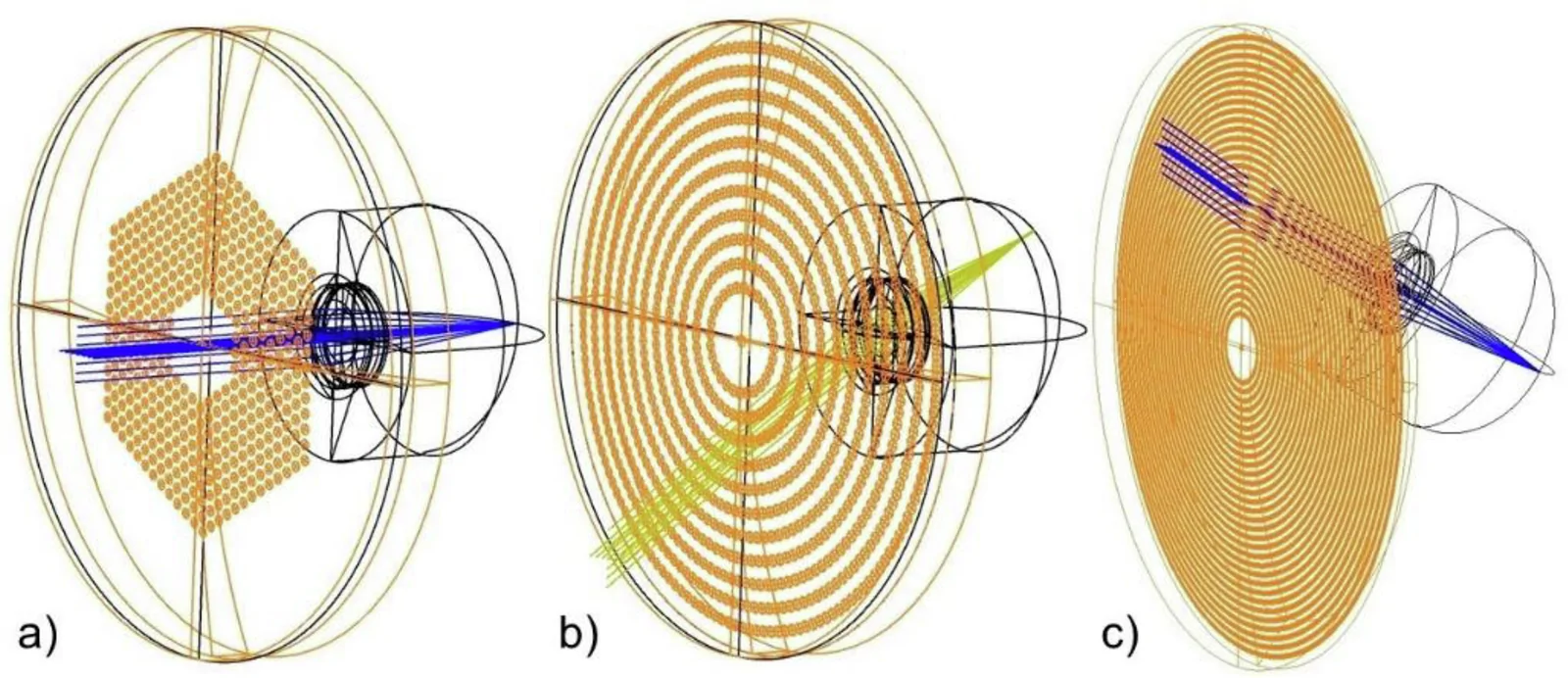
Three myopia control spectacle lenses and viewing conditions. **a** Hoya MiyoSmart (hoyavision.com), with the carrier lens and eye’s optical axes coinciding in central vision. **b** Essilor Stellest (essilor.com), with the carrier lens and eye optical axes coinciding, in peripheral vision (34° field, 3rd ring coinciding with chief ray). **c** Zeiss MyoCare (zeiss.com), with the eye rotating 30° to fixate an off-axis object through the 13th ring. All the lenses have −4.00 D back vertex power and are 12 mm in front of an adult human model eye [171] and 27 mm in front of its centre-of-rotation. Reproduced from Atchison et al. [91].

The intent of these lenses is to provide clear central vision, while imposing myopia control defocus on part of the peripheral field (but see the section “Lenslet-ARray-Integrated (LARI) lens” below). In general, such lenses consist of a carrier lens, whose back vertex power corresponds with the distance correction, on which are superimposed additional optics in part or all of the periphery of one surface. Additional optics can include positive (or negative) power lenslets, phase plates or scattering elements. For the majority of time, a child should be fixating on objects through the centre of the carrier zone, with coincides with the carrier lens’ optical axis and eye’s line-of-sight (Fig. 3a). The proportion of this zone used for foveal vision depends upon pupil size. In peripheral (stationary) vision, the eye continues to fixate through the lens centre, but the object point of interest is in the peripheral field and light travels through the lens periphery (Fig. 3b). Of course, the eye is rarely stationary, and it is also relevant to consider foveal vision when the eye rotates to fixate an object through the lens periphery (Fig. 3c).

Modelling the retinal imagery in the rotating eye situation does not always require much knowledge about the eye’s optical system. Sometimes, only the refraction and the centre-of-rotation position are needed. However, the peripheral situation requires considerable knowledge about the eye’s own optical system, such as biometric details and/or peripheral refraction of the uncorrected eye.

#### DIMS lens, Hoya Corporation

A pioneering lens on which many current lenses are based is the “Defocus Incorporated Multiple segment” (DIMS) lens, developed at The Hong Kong Polytechnic University, and manufactured and marketed by Hoya Corporation under the name MiyoSmart (hoyavision.com). It evolved from the Defocus Incorporated Soft Contact (DISC) contact lens [72]. The first clinical trials were conducted in 2014-2017, the first presentation was at the International Myopia Conference in Birmingham, UK, 2017, and the lens was launched in 2018 in Hong Kong and China (Carly Lam, personal communication). Patents were published in 2018 (China) [73] and 2019 (USA) [74]. The first clinical trial was published in 2019 [75], and to date, at least 13 related research papers have been published by the Hong Kong group [76–88]. In one study [75] the lens showed a mean efficacy in myopic Chinese 8–13-year-old children over a period of 24 months of ≈50% and ≈60% for axial length and myopia, respectively, relative to progression with single vision lenses. These numbers are included because they are reasonably representative of trials with multi-segment lenses.

The MiyoSmart lens is made of polycarbonate, with a true front surface power of 3.36 D. It has 1.0 mm diameter, nominally +3.5 D lenslets on the front surface, although Gantez-Nũnez et al.’s determinations were close to +4 D [89]. The power of each lenslet reduces from the centre to the edge. The arrangement gives nine nested hexagons with the inner and outer boundaries of 9.4 and 33.0 mm, respectively, with centre-to-centre distance between lenslets of 1.5 mm (Fig. 3a). Charman et al. [90] gave a fuller description.

**Fig. 4:**
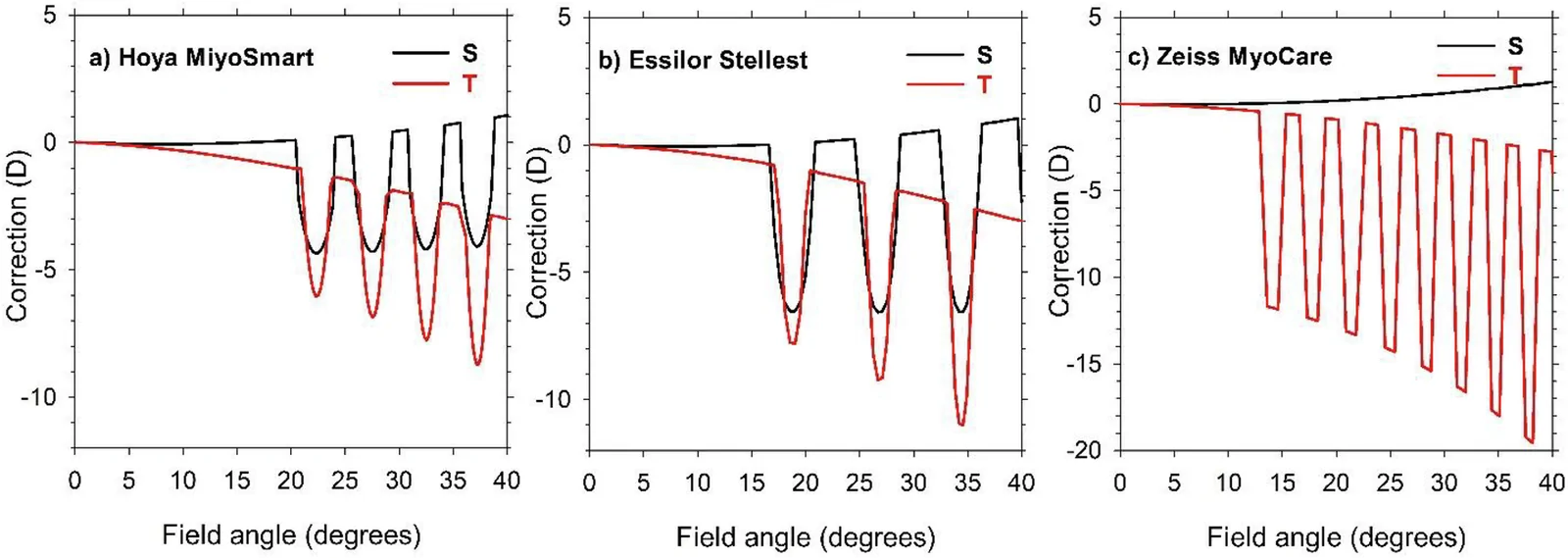
Theoretical sagittal (S) and tangential (T) power corrections of −4 D lenses combined with a model eye along the vertical visual field. **a** Hoya MiyoSmart (hoyavision.com), **b** EssilorLuxottica Stellest (essilor.com) and **c** Zeiss MyoCare (zeiss.com). For the Hoya lens, the vertical meridian is taken to coincide with the apices of the hexagons containing the lenslets. Note the bigger range of the correction scale for the Zeiss MyoCare than for the other lenses. Modified from Atchison et al. [91, 92].

Theoretical imaging and power correction results have been shown for the Hoya MiyoSmart lens for foveal vision with the eye looking through lens centre, peripheral vision with the eye looking through the lens centre and foveal vision with the eye rotated to look through the lens periphery [90–93]. Figure 4a shows the theoretical power corrections in peripheral vision along the vertical visual field for a −4 D MiyoSmart lens, combined with a model eye. Results are also shown for two other lens types (see later). A segment placed at the lens centre would require a correction of −4.0 D to match the additional power of +4.0 D, but at the first hexagon (22°), the correction is considerably greater than this at −4.3 DS/−1.6 DC (mean sphere −5.1 D). This increases to −4.2 DS/−3.6 DC (−6.0 D) at the third hexagon (32.5°). Some parts of the lens near the edge of the segment will be optically unusable, which has been concealed in the figure by ignoring missing results in the plot. This gives an overall picture of “terrible” optics at and near the segments rather than a well-defined “myopic defocus”. This is not necessarily a “bad thing”—see the “Discussion” section.

There is an “enhanced” version of the DIMS spectacle lens, which was named the DIMS TED spectacle lens. It has a reduced central zone of 6.9 mm, increased addition power of 4.5 D and an increased treatment zone diameter of 40 mm. Segment diameter and spacing have not changed. A recent 12-month trial suggests that DIMS TED lens has much higher efficacy than the original DIMS spectacle lens in slowing axial length and myopia progressions, with the following results for axial length change: DIMS TED 0.08 ± 0.02 mm, DIMS 0.21 ± 0.02 mm, single vision lenses 0.35 ± 0.02 mm [94]. DIMS TED spectacle lens is now marketed as Hoya MiyoSmart iQ, while the original DIMS lens is marketed as Hoya MiyoSmart MC.

The Hoya MiyoSmart has inspired similar lenses. One example is the Shanghai Conant Optics MyoEase (conantoptical.com) [95]. This has a regular matrix of segments with a hexagonal outer boundary. Another example is the Defocus distributed multipoint (DDM) lens (Shanghai Weixing Optical Co., Shanghai, China), which, like the DIMS lens, has segments arranged in nine hexagons, but with variable addition power from the three inner hexagons (+3.50 D) to the middle three hexagons (+4.00 D) and to the outer three hexagons (+4.50 D) [96]. It is not clear whether these differences are inspired by theoretical ideas or by efforts to avoid patent restrictions. Further interesting variants are the diversified segmental defocus optimisation (DSDO) Aura 3 and Aura 6 lenses produced by Fitlens Medical Technology Co., Zhuhai, China. These have a 9.5 mm diameter clear area, around which are circular lenslets arranged in 32 radial rows and which reduce in power from about +4 to +2.5 D with increasing distance from the lens centre. Success in myopia control has been demonstrated in a 12-month clinical trial [97]; the lenses appear to influence electrophysiological response of the retina [98].

#### EssilorLuxottica Stellest Lens

The Stellest lens is made of polycarbonate, with a true front surface power of 3.36 D. Lenslets have 1.1 mm diameter and are arranged in 11 rings of diameters 10.0 to 56 mm in 4.6 mm intervals (Fig. 3b). Each segment just touches adjacent segments. Additional powers of the segments, at their centres, in the inner five rings are approximately 5.8 D, but power then decreases to 3.5 D in the outer ring. Lenslets show considerable negative asphericity [91].

EssilorLuxottica refer to the technology of the lens as “Highly Aspherical Lenslet Technology” (H.A.L.T.). Its vision research group has published several papers related to the lens and its variations [42, 99–111]. As well as reductions in the progression of myopia and axial length, the lenses appear to have no significant effect on peripheral light thresholds [101] nor peripheral visual acuity at 22° nasal field [107], they have small to negligible effects on central visual acuity, accommodations and phorias [106], and cause only a small (0.10 logMAR) loss in central visual acuity for a 22° rotating eye [107]. They appear to be able to slow growth in non-myopic children [111] (supported by another research group [112]). Further, they inhibit choroidal thinning [108, 109], (which has been reported also for the Hoya MiyoSmart lens [83]). Significant effects were reported for peripheral eye length (measured along paths initially normal to the anterior cornea) and peripheral refraction [42, 113], with less elongation occurring for the nasal retina as well as for the central retina, with the Stellest lens than for single vision lenses. The “highly aspherical lenslets” lenses were found to be more efficacious than “slightly aspherical lenslets” lenses [99], but the description of the optics indicates that the former have higher overall additions and this may be, at least in part, the reason for the improvement.

Figure 4b shows theoretical power corrections in the peripheral vision for a −4 D Essilor Stellest lens, combined with the model eye. What has been described for the Hoya MiyoSmart is increased by about 30% for the Stellest lens, e.g., at the third ring (34.4°), the correction is −6.5 DS/−4.5 DC (mean sphere −8.7 D).

A new version, called the Stellest 2.0, using “H.A.L.T. MAX” technology, has about twice the power and asphericity (in terms of the drop in lenslet power from the centre to the edges). It has been claimed to slow the progression of axial length further. In a cross-over trial with lenses worn on opposite eyes and then switched over, increases in axial length over the first 6 months were 0.11 ± 0.02 and 0.04 ± 0.02 mm for Stellest and Stellest 2.0 lenses, respectively. Corresponding increases over the second 6 months after cross-over were 0.12 ± 0.01 and 0.08 ± 0.01 mm [103].

#### Asymmetric Multipoint Defocus Technique (AMDT) Lens, Mingyue Optical Lens Co (mingyue.com)

**Fig. 5:**
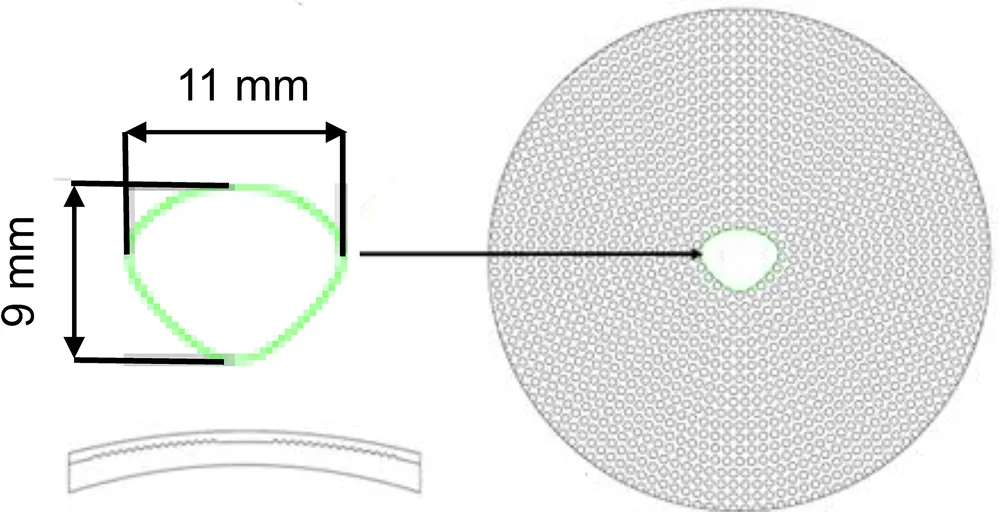
Layout of the Asymmetric Multipoint Defocus Technique (AMDT) lens. Modified from Wang et al. [114].

The AMDT lens design has a curvilinear triangle-shaped distance optical zone having height and width of 9 and 11 mm, respectively (Fig. 5). A total of 1329 + 5.00 D lenslets (1.2 mm diameter) placed 1.7 mm apart are arranged in a concentric ring pattern covering the rest of the lens. Rather than being placed on the front surface, the microlenses are positioned in a middle layer of the lens, using an increased refractive index to provide their power [114].

#### Lenslet-ARray-Integrated (LARI) lens (Shanghai iSparX Medical, Shanghai, China)

**Fig. 6:**
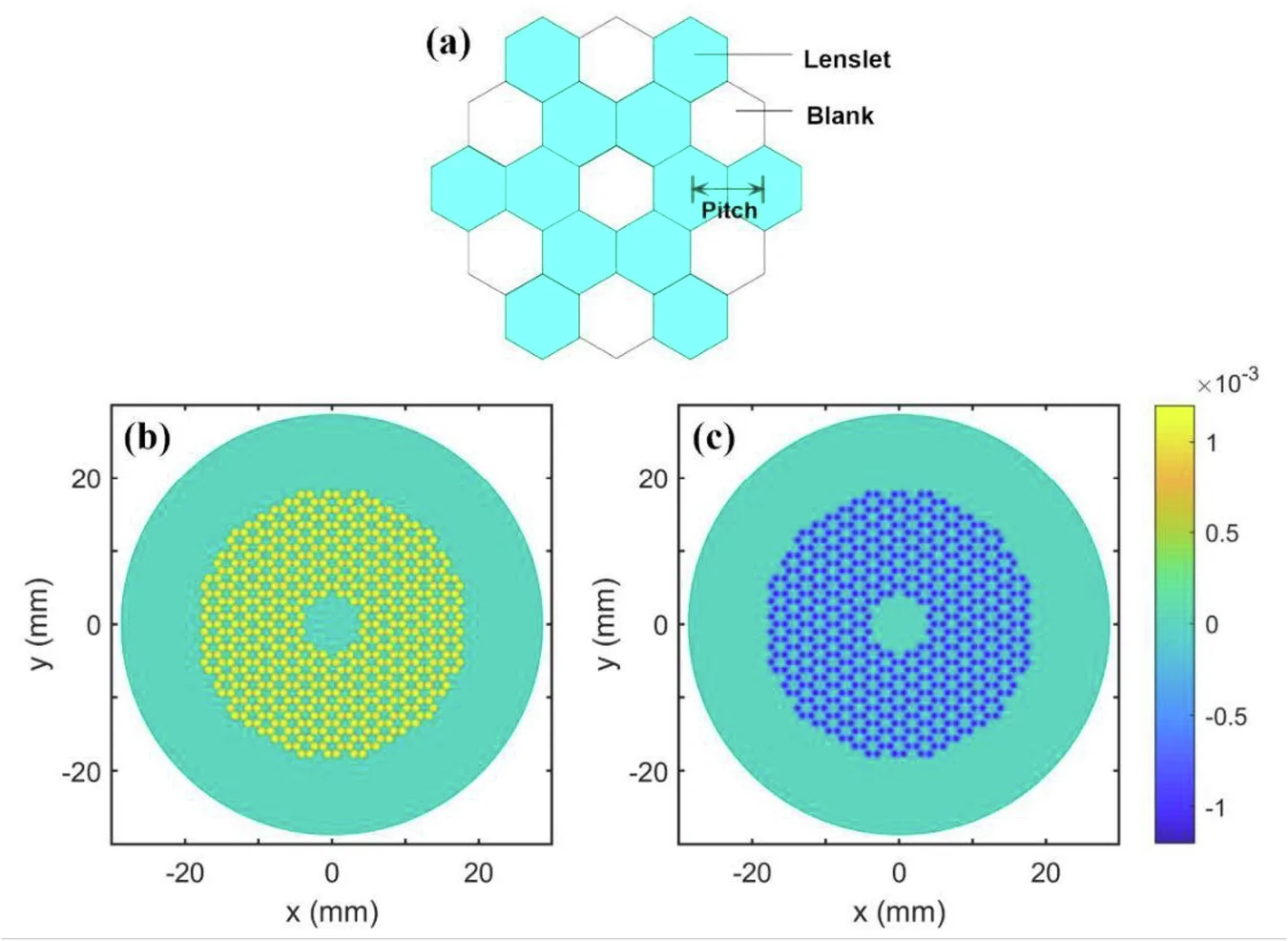
Structure of the Lenslet-ARray-Integrated (LARI) lenses. **a** shows the background (“blank”) and lenslets distributions, while **b** shows the positive LARI (PLARI) version and **c** shows the negative LARI (NLARI) version. The scale shows sagittal heights of the segments relative to the base curve. Reproduced from Jiang et al. [116].

The lens is based on a Chinese patent [115]. Jiang et al. [116] described the lens structure (Fig. 6). The lenses are made of polycarbonate and have a true front surface power of +2.32 D, with a central distance zone of about 8 mm. The two versions have additional power of +3.0 D (positive LARI, PLARI) and −3.0 D (negative LARI, NLARI). The lenslets have a hexagonal shape with a pitch of 1.2 mm (each side has a length of ~0.7 mm) and extend to ~36 mm in diameter. Su et al. [43, 44] described clinical trials that produced the surprising results that the two versions were equally effective in slowing myopia progression and axial length elongation; this is mentioned further in the section “Treatment success”.

A further development of these ideas is the high-order phase element (HOPE) spectacle lens [116]. The lenslets of the LARI lens, which can each be considered as rotationally-symmetric phase delaying devices, producing wavefronts with second-order aberrations, are replaced by phase elements with more complex symmetry and other properties which can generate wavefronts with higher-order aberrations. This allows the design of a whole family of HOPE designs, each having slightly different properties. Although the exact effects depend upon the conditions of use, the higher-order aberrated wavefronts from all of these lenses produce a modulation transfer function in the carrier focus plane which falls rapidly with spatial frequency to reach a low value at about 10 c/degree, and remains low at medium and high spatial frequencies. The corresponding diffuse blur disc diameter is about 3°. Low contrast of associated retinal images is thought to be responsible for the myopia control effect.

#### Individualised Ocular Refraction Customisation (IORC) Lens, Shenzhen Thondar (thondar.com)

This lens has the interesting feature that the back surface is designed to correct the relative peripheral refraction of individual patients. This is done after two-dimensional relative peripheral refraction measurement with the company’s Multispectral Refraction Topography instrument [117], which, to the authors’ knowledge, has not been compared against other instruments such as those using Hartmann–Shack technology. This has the same aim, but a different approach from the DIMS2 lens mentioned above in maintaining a constant effective addition throughout the visual field. Given the compromises that are made in sophisticated lenses such as progressive addition lenses, where unwanted astigmatism is produced and must be “pushed” to less-used regions of the lenses, it is not clear how well correction can be made across a two-dimensional visual field out to 53° diameter.

The lens has 1.0 mm diameter segments arranged in rings from ~9 to 38 mm diameters. A clinical trial has been reported [118, 119], with a high addition version (IORC-H, +4.50) having higher efficacy than medium (M) and low (L) addition versions (IORC-M, +3.50 D; IORC-L, +2.50 D).

### Annular Cylinder Lenses

This family of lenses includes several concentric, narrow annular refracting elements, having nominally zero power circumferentially and full cylindrical power in the radial direction.

#### MyoCare lens, Carl Zeiss Vision (zeiss.com)

MyoCare was designed based on licences from Wenzhou Medical University and Hong Kong Polytechnic patents. It was launched first in 2022 in China, followed soon after in the rest of the world. A few clinical trials have been published to date [120–124].

MyoCare lenses (Fig. 3c) are made of polycarbonate, with a true front surface power of 2.31 D. They have cylindrical annular rings (C.A.R.E.). The rings have their full power at right angles to the ring borders (i.e., radially) but have no power along the annuli (circumferentially). Each of the ~30 annuli has a width of 0.5 mm and is 0.5 mm from the adjacent annulus. There are two variants. “MyoCare” is for younger children, with a central distance zone of 7.0 mm and a mean addition (half the added cylinder) of +4.6 D. The centres of the ring elements have semi-diameters of 3.75, 4.75 mm, etc., up to 33.75 mm for the 31st ring. “MyoCare S” is for older children, with a central zone of 9.0 mm and a +3.8 D addition [65, 125].

Figure 4c shows the theoretical power corrections in peripheral vision for a −4 D MyoCare lens, combined with the model eye. There is no sagittal power contribution to the correction through the ring, but apart from this, the lens produces much higher refractions than those for the Hoya MiyoSmart and EssilorLuxottica Stellest lenses, e.g., at 34.8° (7th ring) correction is +0.9 DS/−18.6 DC; the mean sphere of −8.8 D is twice that which would occur if a ring could be placed near the centre of the lens.

The newer Zeiss MyoEase or Tamer lens has only four broad cylindrical annuli of +4 D mean power, with a width of about 1.3 mm and diameters of approximately 8, 14, 20 and 26 mm, respectively, while the carrier has additional positive power produced by reducing (negative) back surface power in its periphery, so that it presumably attempts to achieve myopia control through a combination of multisegment design and relative myopic peripheral refraction of the carrier. It is currently available only in China.

The latest Zeiss lens is the MyoActive, based on a study of five designs with interesting features [126]. In appearance, it looks like the MyoCare, but the power in each ring is variable (1.5–5.0 D mean sphere), and the power at a point in any ring does not align with the same power in adjacent rings, presumably to counteract any adaptation that might work against slowing myopia progression.

### DOT technology

As outlined earlier (see Theories), the image contrast (contrast modulation) theory suggests that myopia can arise as a result of excessive contrast in the image falling on the mid-peripheral retina. To combat this, diffusion optics technology (DOT) treatment involves the use of spectacle lenses with many small scattering regions to reduce image contrast on the peripheral retina [127]. Scattering is wide-angle [128]. Neitz and Neitz [47] stated “A uniform plane of scattered light is superimposed on images formed by the background lens, lowering contrast slightly but uniformly”. The image contrast in the periphery would already be considerably reduced due to substantial second- and higher-order aberrations of the eye (see Fig. 1).

#### Diffusion Optics Technology (sightglassvision.com)

While relevant papers appeared only in 2023, there has been a considerable period of development, with the first related US patent appearing in 2017 [129]. The lens has a clear distance zone of 5 mm diameter, which is smaller than that of other lenses described here. Beyond that, there are small scattering regions (diffusers) described as “having a diameter of ~0.14 mm and a height of ~ 0.2 mm” [128], which reduce the higher spatial frequency range that is transmitted. Reports have been presented at 12 months and 4 years into a clinical trial, with the commercial product (DOT 0.2) with 0.365 mm spacing of diffusers performing better than a variant with 0.240 mm spacing [128, 130]. This was attributed to a higher drop-out rate for the denser lens, caused by dislike of lens appearance and adaptation difficulty [130].

The loss of visual acuity in comparison with the central value, when looking through the periphery of the lenses, is low (<0.05 high-contrast logMAR) [131] and therefore the lens wearer would be likely to use eye rotation frequently when fixating objects. Thus, there may be a role for central as well as peripheral vision in the treatment. Surprisingly, as yet no measurements of the angular characteristics of the scattering produced by the scattering elements have been published.

#### Diffusion optics multiple segments (DOMS) internacional.com.ar/optico/producto/majestic-myopia

A clear 9-mm-diameter zone is surrounded by a treatment zone having 1.0 mm diffusers separated by 0.75 mm, and extending out to 36 mm diameter [132, 133].

#### Other

A recent suggestion is the use of spectacle lenses with small, non-refractive opaque features on their surface. If small, such features would be expected to scatter and diffract light and lower the contrast of the retinal image in a similar way to that of DOT technology lenses. Some developmental work has been carried out [134].

Complementary treatment might well include efforts to give children greater exposure to more natural environments, with their lower contrasts.

### Concentric, or rotationally symmetrical, bifocals

Opulens (Novar Technologies novar-tech.com) produces a concentric bifocal called “Myofix” [135]. A central distance zone of 9 mm is surrounded by a short transition zone and then by a zone with a +3.50 D addition on the back surface. Atchison and Charman [56] determined theoretical optical performance; the addition starts at about 23° field for a −4 D lens at 12 mm vertex distance, and beyond 30° the correction becomes increasingly greater than the specified 3.5 D. A 12-month clinical trial has been completed recently [136].

Optocare MyOnic (optocare.com.au) has a central distance zone of 10 mm, surrounded by a transition zone, whose power increases to +2.00 or +3.00 D, and after which the power is constant [95].

## Treatment success

There has been an explosion in clinical trials over the last 5 years or so, particularly concerning spectacle lens treatments. However, the section here is small as treatment success has been covered in several reviews published in 2025 and 2026 [137–146]. Some of these reviews discussed methodology, such as parallel-group, cross-over trials, paired-eye type (different treatments to the two eyes of participants) and cluster-randomised (randomisation of groups of participants rather than individual participants). Further, the effect of the COVID-19 pandemic lockdowns on some trials covering the 2020–2021 period was discussed. Reported outcomes are slowing of myopia and axial length progression relative to control groups (e.g., single-vision spectacles). Brennan et al. [147] argued that treatment success should be measured in absolute rather than relative terms and Wolffsohn et al. [148] provided guidelines for clinical trials.

Considering recent reviews and in particular that of Bullimore et al. [140], our estimates for the efficacies of short-term treatments, of the order of 12 months, for myopia progression are approximately as follows: spectacles and contact lenses 50%, orthokeratology 60% and red-light therapy 80%+. It must be realised that there is a wide range of values within treatment groups. The reviews had slightly different grouping systems. Bullimore et al. presented a useful figure of the slowing of axial elongation against time for various trials of spectacles, contact lenses, orthokeratology, red light therapy and atropine concentrations of 0.01% and higher. While red light therapy comes out as the most successful, there are safety concerns and treatment cessation is associated with significant rebound [140, 149]. In their meta-analysis of myopia-controlling spectacle lens interventions, Perea-Romero et al. [143] concluded that although such lenses show short-term benefits, their long-term efficacy remains uncertain, with the analysis showing no significant control effects on changes in axial growth or spherical refractive error after 24 months. Lawrenson et al. [137] were cautious regarding evidence for 2 years and beyond and wrote: “uncertainty remains about the sustained effect of these interventions”. Bullimore et al. [140] were more enthusiastic about results persisting after the first year, while Lam et al. claimed that DIMS (MiyoSmart, hoyavision.com) spectacles provided sustained myopia control without adverse effects over a 6-year period [76].

Rebound refers to progression that increases following treatment cessation that is faster than expected in an age-matched control group [150]. Bullimore and Brennan [151] found no evidence for rebound for spectacles and soft contact lenses, but there was rebound for atropine and red light therapy.

Treatment success should be concerned with intertwining factors of adaptation, compliance and safety. The cost of treatment should also be considered. For multisegment and DOT lenses, visual functions are affected little compared with wearing single-vision lenses [76, 99, 130, 131, 152], which makes for easy adaptation and tolerance. Without careful investigation, myopia-control spectacle lenses do not look different from single vision lenses. Trial compliance has been reported as high [76, 120, 130]. Spectacle lenses are inherently safe, provided they are made from impact-resistant materials such as polycarbonate. The cost of a pair of spectacles may be several hundred Australian dollars, but it is likely to come down with the proliferation of new manufacturers.

Some consideration has been made regarding interventions in non-emmetropes at risk of developing myopia (i.e., “pre-myopes”). Trials in Chinese children have found multisegment lenses to be effective, relative to single vision [153], plano lenses [154], no lenses [154, 155] or pre-treatment rates [106], at slowing axial elongation and refractive change [111, 112].

## Discussion

A wide range of approaches for the control of myopia development and progression is currently available and many of these have proven to be effective. However, in many cases of “myopia-controlling” lenses, it is not clear why the manufacturer has incorporated their specific design features, and it may be speculated that, in a potentially lucrative field, marketing and the need to avoid patent infringements may have sometimes taken precedence over careful studies to establish which parameters and values are most important. Thus, having established the basic efficacy of many designs, efforts are only now being made to optimise their efficacy by adjusting various design parameters.

In the area of multisegment lenses, this optimisation process has mainly involved increasing the power and asphericity of the lenslets used. As discussed earlier, the Hoya MiyoSmart has been developed into the MiyoSmart iQ, with a reduced central zone, increased addition power and an increased treatment zone. Similarly, the H.A.L.T. technology of the earlier Stellest lens has been replaced by H.A.L.T. MAX with lenslets of greater power and asphericity, with a trial of the latter showing better myopia control than its predecessor [103]. The combination of high power and asphericity would be expected to replace a well-defined “myopic focus” formed by each lenslet with a longitudinally extended volume of poor myopic focus, with each zonal focus point being surrounded by an extensive halo [103]. Assuming that accommodation always brings the image formed by the carrier lens onto the retina, then the combined effect of all the lenslets within the aperture of the image-forming beam would be to reduce the contrast and high spatial frequency content of this carrier image [93]. The other aspect of multisegment and DOT design under consideration is the diameter of the clear central optical zone, which provides optimal acuity. This ranges from 9 mm for the MyoCare S to 5 mm for DOT lenses. The dimensions of the roughly triangular clear area of the AMTD lens (about 10 mm, Fig. 5) are based on an eye tracker study which recorded the position of the centre of the pupil with respect to the lens during near and distant fixation [156]. Swiaztczak et al. [157] suggested that the retina is most sensitive to defocusing the annular area 3 to 9 degrees from fixation. On this basis, current central clear optical zone diameters might benefit from a slight reduction.

This suggests that, with minor refinements, current multisegment lenses are behaving in the dual-focus, simultaneous-vision way that was originally suggested, with additional myopic defocus being provided by the lenslets, inhibiting increases in axial length and myopia. Yet if myopic defocus is desirable, then why do LARI spectacle lenses with negatively powered lenslets have the same effects on myopia progression as those with positive lenslets [43, 44]? Moreover, the outer zones of some nominally positive-powered lenslets produce hyperopic rather than myopic defocus [158–161]. These results cast doubt on the simple dual-focus explanation for the myopia-control effect of these lenses. Importantly, the results of modelling the off-axis optical performance of multisegment-spectacle/eye systems show that in the peripheral field, the combined effects of the lens aberrations and the eye heavily degrade the retinal images. Under these conditions, the lenses can be considered equivalent to spatial frequency filters, which reduce the medium and high spatial frequency content in the mid-peripheral retinal image. These and other considerations have led some authors to suggest that the multisegment spectacle (and contact lenses) operate via contrast reduction, similar to DOT lenses, rather than through a dual focus effect [162, 163].

Clearly, more work is required to clarify these issues if the true mechanisms of myopia control are to be properly understood. One problem in modelling lens optical performance is the limited information released by manufacturers on the details of their designs, e.g., manufacturing techniques used, lenslet asphericities and the nature and effects of any hard or other coatings applied to multisegment lenses, as well as details of the fill factor and angular light-scattering characteristics of the elements of DOT lenses. Further laboratory work on the optical details of many of these lenses is required if their actions are to be fully understood. It may be that the use of instrumentation allowing experimental simulation of the images produced by simultaneous (dual-focus) vision lenses will be helpful [164].

## Conclusion

The last decade and a half have seen exciting progress in the development of optical methods for myopia control, so that some methods have now become safe, valuable tools well suited for integration into routine optometric practice. Although some myopia-control spectacles (and contact lenses) were originally designed on the basis of different hypothetical control mechanisms, evidence is mounting that reduced modulation transfer at medium and high spatial frequencies of images in the peripheral retina may be a common factor in their action. Further development should help to clarify the exact nature of the mechanisms of action of the different lenses and improve their efficacy.

## Data Availability

No datasets were generated or analysed during the current study.
